# Raus aus der Neueinstellungskrise!

**DOI:** 10.1007/s10273-020-2698-z

**Published:** 2020-07-18

**Authors:** Christian Merkl, Enzo Weber

**Affiliations:** 1 FB Wirtschafts- und Sozialwissenschaften, Friedrich-Alexander-Universität Erlangen-Nürnberg, Lange Gasse 20, 90403 Nürnberg, Deutschland; 2 Forschungsbereich A1, Inst. f. Arbeitsmarkt- und Berufsforschung, Regensburger Str. 100, 90478 Nürnberg, Deutschland

**Keywords:** J08, J23, H53

## Abstract

Anders als in der Wirtschafts- und Finanzkrise 2009 ist der Arbeitsmarkt in der Corona-Krise 2020 deutlich stärker betroffen. Insbesondere die Neueinstellungen sind sehr stark zurückgegangen — mit der Gefahr, dass daraus eine langwierige Arbeitslosigkeit und eine „Generation Corona“ der Absolventen entsteht. Um diesem Phänomen frühzeitig entgegenzutreten, wären temporäre Einstellungszuschüsse auch unter Einbeziehung aller Risiken eine sinnvolle Maßnahme.

## Abstract (en)

In contrast to the economic and financial crisis of 2009, the labour market has been hit harder during the coronavirus crisis in 2020. New hires have declined substantially, which may lead to persistent unemployment and a “generation COVID-19” of graduates. Employment subsidies would be a suitable measure to stabilise the economy and the labour market and to reduce these risks at an early stage.

Im Jahr 2009 stürzte Deutschland in eine tiefe Rezession, aber die Katastrophe am Arbeitsmarkt blieb glücklicherweise aus. 2020 wird es aber keine Wiederholung des damaligen Drehbuchs geben. Denn der Arbeitsmarkt wurde nicht nur durch Kurzarbeit gut durch die Finanzkrise gebracht, sondern auch durch relativ stabile Neueinstellungen. Nach IAB-Stellenerhebung gingen die Einstellungen 2009 um weniger als 10 % gegenüber dem Vorjahr zurück (Bossler et al., 2017). Seit März 2020 sind die Abgänge aus Arbeitslosigkeit in Beschäftigung und die Meldungen neuer Stellen dagegen drastisch eingebrochen. Abbildung 1 zeigt die Abgangsrate und den Stellenbestand. Auch die Suchintensität am Arbeitsmarkt nahm stark ab (Hartl et al., 2020).

Corona droht zur Neueinstellungskrise zu werden. So wie das Kurzarbeitergeld Betriebe dabei unterstützt, Entlassungen zu vermeiden, ist deshalb dringend auch eine Förderung von Neueinstellungen erforderlich. Von diesen gibt es normalerweise etliche Millionen pro Jahr – wenn auch nur ein Teil ausbleibt, sind langwierige Arbeitslosigkeit und eine „Generation Corona" der Absolventen vorprogrammiert (Hutter und Weber, 2020). Damit verbundene Risiken sind insbesondere Hysterese und bleibende Scarring-Effekte (Möller und Umkehrer 2015) bei Berufseinsteigern.

## Größte Rezession der Nachkriegsgeschichte

Laut aktuellen BIP-Prognosen erlebt Deutschland 2020 die schwerste Rezession der deutschen Nachkriegsgeschichte. Die aktuellen Zahlen für Neueinstellungen, offene Stellen und Beschäftigung zeigen, dass der deutsche Arbeitsmarkt wesentlich mehr in Mitleidenschaft gezogen wird als in der Rezession der Jahre 2008/2009. Während Entlassungen auch in der 2020er Rezession durch Kurzarbeit stabilisiert werden, ergeben sich deutliche Unterschiede bei den Neueinstellungen. Die zugrundeliegenden Ursachen werden sicher ein wichtiges Thema in der künftigen makroökonomischen Forschung sein. Schon jetzt ist aber klar, dass es während der Finanzkrise der Jahre 2008/2009 Sonderfaktoren gab, die 2020 in der Form nicht vorhanden sind. So argumentieren Burda und Hunt (2011), dass Unternehmen im Aufschwung der Jahre 2005 bis 2007 die Wirtschaftslage zu pessimistisch eingeschätzt hatten, zu wenig neue Arbeitnehmer eingestellt hatten und folglich der Personalanpassungsbedarf 2009 gering war. Boysen-Hogrefe und Groll (2010) argumentieren, dass deutsche Unternehmen während der Finanzkrise sehr stark von den vorherigen moderaten Lohnentwicklung profitierten, die zu einem deutlichen Anstieg der Arbeitsnachfrage führte und damit den rezessiven Schock auf dem Arbeitsmarkt dämpfte. Gehrke et al. (2019) sowie Klein und Schimann (2019) stellen einen Zusammenhang zwischen den Hartz-Reformen, dem daraus resultierenden Arbeitsmarktaufschwung und dem milden Verlauf am deutschen Arbeitsmarkt im Jahr 2009 her. Nach den Ergebnissen von Hutter et al. (2019) und Klinger und Weber (2016) wurden die Einstellungen in der

* Eine Kurzversion des vorliegenden Artikels erschien am 30. Juni 2020 in der Frankfurter Allgemeinen Zeitung.

**Prof. Dr. Christian Merkl** ist Professor für Makroökonomik an der Friedrich-Alexander-Universität Erlangen-Nürnberg und Forschungsprofessor am Institut für Arbeitsmarkt- und Berufsforschung (IAB).

**Prof. Dr. Enzo Weber** ist Leiter des Forschungsbereichs „Prognosen und gesamtwirtschaftliche Analysen" am IAB und Professor für Empirische Wirtschaftsforschung, insbesondere Makroökonometrie und Arbeitsmarkt, an der Universität Regensburg.

Abbildung 1

**Abgangsrate aus Arbeitslosigkeit, gemeldete Stellen[1]**

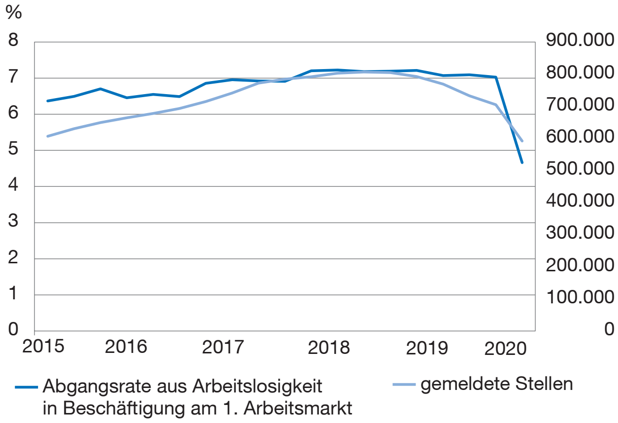

[1] Durchschnittliche monatliche Abgangsrate aus Arbeitslosigkeit auf Quartalsfrequenz und Bestand offener Stellen.

Quelle: Statistik der Bundesagentur für Arbeit.

Krise durch positive Trends bei der Effizienz des Matchings und der Intensität der Stellenschaffung gestützt. Unabhängig von der quantitativen Bedeutung der einzelnen Sonderfaktoren führten diese zu einem geringeren Rückgang des Arbeitskräftebedarfs 2009 als 2020. Folglich war die Notwendigkeit geringer, die Beschäftigung nach unten anzupassen, wie dies derzeit vor allem über weniger Neueinstellungen passiert. Vor diesem Hintergrund sind Einstellungszuschüsse das folgerichtige Gegenmittel, um Unternehmen den Anreiz zu geben, mehr einzustellen.

## Studien zeigen positive Wirkungen

Es gibt gute und wissenschaftlich abgesicherte Argumente für eine solche temporäre Einstellungsförderung in der jetzigen Krise. So zeigt eine hochrangig veröffentlichte empirische Studie (Cahuc et al., 2018a), dass ein vorübergehender Einstellungszuschuss im Krisenjahr 2009 in Frankreich erhebliche positive Beschäftigungseffekte entfaltete. Eine makroökonomische Simulationsstudie für Deutschland ergibt darüber hinaus, dass ein Einstellungszuschuss Beschäftigung und Bruttoinlandsprodukt wesentlich stärker erhöhen würde als die gleichen Bruttoausgaben für traditionelle Staatsausgabenprogramme (Faia et al., 2013). Der Multiplikatoreffekt ist bedeutend größer, weil direkt Jobs und Einkommen geschaffen werden.

Temporäre und unerwartete Einstellungszuschüsse würden das Bruttolohnniveau unmittelbar kaum verändern (Stephan, 2010), da Regeln wie Tarifverträge und Mindestlöhne den Rahmen setzen. Folglich würden diese zu einem großen Teil bei den einstellenden Arbeitgebern ankommen und entsprechend die Einstellungsanreize stark erhöhen.

Neben den gesamtwirtschaftlichen Ergebnissen zeigt eine Reihe von Evaluationsstudien für Deutschland positive Wirkungen von Einstellungszuschüssen, die auf bestimmte Gruppen fokussiert sind. Mikroökonometrische Schätzungen (z. B. Bernhard et al., 2008; und Jirjahn et al., 2009) und die Simulation verschiedener Interventionen (Brown et al., 2011) liefern diesbezüglich kohärente Ergebnisse.

## Mitnahmeeffekte zu befürchten?

In der wirtschaftspolitischen Debatte werden vor allem Mitnahmeeffekte als Argument gegen Einstellungszuschüsse vorgebracht. Natürlich wäre es unvermeidlich, dass der Einstellungszuschuss auch in vielen Fällen bezahlt würde, in denen die Einstellung ohnehin erfolgt wäre. Interessanterweise wurden ähnliche Argumente bei anderen Maßnahmen wie der geplanten Mehrwertsteuersenkung kaum vorgebracht (Konsum, der sowieso stattfinden würde). Makroökonomische Simulationen für Deutschland zeigen, dass die Mitnahmeeffekte bei einer temporären Mehrwertsteuersenkung größer sind als bei temporären Einstellungszuschüssen (Faia et al., 2010).

Es geht hier keineswegs darum, gegen eine Mehrwertsteuersenkung zu argumentieren, die durchaus auch ihre Vorzüge hat. Sondern es geht darum, dass Einstellungszuschüsse den Arbeitsmarkt sehr effektiv aus der Krise bringen könnten. Die empirische Studie für Frankreich liefert starke Argumente für eine hohe Kosteneffektivität von Einstellungszuschüssen. Sie findet, dass alleine die eingesparten Sozialausgaben die Bruttokosten des Zuschusses kompensieren (siehe Cahuc et al., 2018a, b). Die Wirksamkeit von Einstellungszuschüssen zahlt sich also auch fiskalisch aus. Eine Einstellungsförderung sollte in der gegenwärtigen Situation möglichst einfach und umfassend ausgestaltet sein. Eine praktikable Lösung bietet der als „Rettungsschirm für Neueinstellungen" (Weber, 2020) diskutierte Vorschlag, innerhalb eines bestimmtes Zeitraums bei neuen Jobs auf die Zahlung von Sozialbeiträgen durch die Arbeitgeber zu verzichten und diese stattdessen aus dem Bundeshaushalt zu finanzieren. Bei Arbeitgeberbeiträgen von rund 21 % würde das erhebliche finanzielle Anreize setzen. Dies würde insbesondere für frühzeitige Einstellungen gelten, die entsprechend länger von der monatlichen Förderung profitieren.

## Was kostet der Einstellungszuschuss?

Wir berechnen die Bruttokosten für den Einstellungszuschuss auf Basis des Vorkrisenniveaus von 8 Mio. Neueinstellungen pro Jahr aus den Integrierten Erwerbsbiographien des IAB. Da die Berechnung auf Basis der Neueinstellungen im vergangenen Jahr erfolgt, handelt es sich vor dem Hintergrund des Rückgangs der Neueinstellungen in diesem Jahr sicherlich um eine Obergrenze für die Kosten. Bei unserer Berechnung gehen wir davon aus, dass die Neueinstellungen monatlich gleichverteilt für das Jahr erfolgen. Der durchschnittliche Lohn bei Einstellung (mit Kappung an der Beitragsbemessungsgrenze) wird mit 2292 Euro aus der IAB-Stellenerhebung errechnet. Die Förderung wird nur für Jobs relevant, die länger als sechs Monate dauern. Die Verteilung der Dauer sozialversicherungspflichtiger Beschäftigungsverhältnisse entnehmen wir der Statistik der Bundesagentur für Arbeit. Wir nehmen dabei an, dass alle Beschäftigungsverhältnisse ab einer Dauer von drei Monaten verlängert werden, um von der Förderung zu profitieren. Unter dieser Annahme würden 74 % der Neueinstellungen eine Förderung erhalten. Die Bruttokosten für ein halbes Jahr lägen dann bei knapp 5 Mrd. Euro. Den Bruttokosten stehen eingesparte Sozialausgaben sowie zusätzliche Steuer- und Beitragseinnahmen gegenüber.

Wie bei jeder staatlichen Maßnahme sind unerwünschte Nebenwirkungen zu bedenken, insbesondere mögliche Drehtüreffekte. Diese lassen sich durch einfache Regeln stark begrenzen, indem etwa Fälle ausgeschlossen werden, in denen nach Bekanntwerden der Förderung ein Beschäftigungsverhältnis der betreffenden Person beim selben Arbeitgeber beendet wurde. Ein sinnvolles Kriterium wäre auch, dass das geförderte Beschäftigungsverhältnis länger als sechs Monate (die übliche Probezeit) dauert. Das würde zugleich Fälle ausschließen, in denen Unternehmen ihre Beschäftigten in kurzfristigen Jobs austauschen, um Sozialversicherungsbeiträge zu sparen. Derartige Regeln sind bei existierender Einstellungsförderung schwer vermittelbarer Personen bereits erprobt, bei der sich kaum unerwünschte Nebeneffekte zeigen (z. B. Moczall, 2013). Dies gilt ebenso für den französischen Einstellungszuschuss (Cahuc et al., 2018a).

Die Risiken einer anhaltenden Neueinstellungskrise in Deutschland sind gravierend. In den vergangenen Monaten gab es bereits einen massiven Rückgang der Einstellungen aus Arbeitslosigkeit. Mitnahmeeffekte bei Einstellungszuschüssen wären derzeit also so gering und die Kosteneffektivität damit so hoch wie lange nicht mehr. Ein erster Schritt in diese Richtung sind die Prämien für die Einstellung von Auszubildenden im Corona-Konjunkturpaket. Es ist aber auch für den gesamten Arbeitsmarkt die richtige Zeit für Einstellungszuschüsse!
